# The Breadth of Viruses in Human Semen

**DOI:** 10.3201/eid2311.171049

**Published:** 2017-11

**Authors:** Alex P. Salam, Peter W. Horby

**Affiliations:** University of Oxford, Oxford, UK

**Keywords:** semen, sexual transmission, viruses, Zika, Ebola, epidemic, sperm, spermatozoa

## Abstract

Zika virus RNA is frequently detected in the semen of men after Zika virus infection. To learn more about persistence of viruses in genital fluids, we searched PubMed for relevant articles. We found evidence that 27 viruses, across a broad range of virus families, can be found in human semen.

The finding by Atkinson et al. that Zika virus RNA is frequently detected in the semen of men after infection (*1*) highlights our knowledge gaps regarding the persistence of viruses in genital fluids, especially semen. Replicating Zika virus (*2*), like Ebola and Marburg viruses (*3*), has been isolated from semen and has been sexually transmitted. However, it is probable that many more viruses capable of causing viremia (presence of virus in the blood) can be found in semen. Seeding to the male reproductive tract may frequently occur in the context of viremia because the blood–testes/deferens/epididymis barriers are imperfect barriers to viruses, especially in the presence of systemic or local inflammation (*4*). Virus may persist even if incapable of replicating within the male reproductive tract because the testes are immunologically privileged (*4*); that is, within the testes, the immune response is restricted to enable the survival of sperm, which are immunogenic. Virus may also be transmitted to semen as a result of survival and replication within the accessory glands (*5*).

To investigate the breadth of viruses in semen, we performed a PubMed search by using the terms “virus* AND semen OR sperm* OR seminal.” We imposed no date or language restrictions. This search returned 3,818 results. We screened the titles, abstracts, and full text articles for data that described detection of viruses in semen by nucleic acid amplification or detection, antigen detection, replication in cell culture, or replication in an animal system. We restricted the results to viruses capable of causing viremia. Where we found evidence for virus in semen, we then searched PubMed for evidence of sexual transmission by using the terms “(name of virus) AND sex* AND Transm*.” 

Our search revealed that 27 viruses that can result in viremia have been found in human semen (Table). For many of these, data on sexual transmission are lacking. Of these 27 viruses, many cause chronic or latent infection (e.g., HIV virus, cytomegalovirus). However, several cause acute infections, including Lassa fever, Rift Valley fever, and chikungunya viruses. Of those causing acute infections, only Zika and Ebola viruses have been systematically screened for in semen (i.e., in case series or cohort studies rather than case reports). These 27 viruses come from diverse families, suggesting that the presence of many viruses in semen is unlikely to be exclusively dependent on specific or conserved viral epitopes, ability of virus to replicate within the male reproductive tract, or common mechanisms of immune evasion. Other factors that may also influence whether viruses exist in semen are level of viremia, inflammatory mediators (altering blood-barrier permeability), systemic immunosuppression, male reproductive tract immune responses, presence of sexually transmitted diseases, and virus structural stability. In mammals, numerous viruses are detectable in semen, including viruses that can cause disease in humans, such as Japanese encephalitis virus, foot and mouth disease virus, parainfluenza virus, and paravaccinia virus (*6*). Several other viruses that result in viremia can cause orchitis and have been detected in human testes, suggesting the possibility that these viruses may also be detectable in semen. These viruses include influenza virus, lymphocytic choriomeningitis virus, phlebotomus fever virus, cocksackie B virus, echovirus, dengue virus, systemic acute respiratory syndrome virus, parvovirus, smallpox virus, vaccinia virus, and rubella virus (*7*).

**Table:** Viruses that are capable of causing viremia and found in human semen*

Virus	Family	Detection in semen, maximum detection time, d	Isolation from semen, maximum detection time, d	Evidence for sexual transmission within same cohort
Adenoviruses	Adenoviridae	AD	RCC	Unknown
Transfusion transmitted virus	Anelloviridae	NAA	No data found	Unknown
Lassa fever virus†	Arenaviridae	NAA, 103	RCC, 20	Unknown
Rift Valley fever virus†	Bunyaviridae	NAA, 117	No data found	Unknown
Ebola virus	Filoviridae	NAA, 531	RCC, 82	Epi + mol + sem
Marburg virus†	Filoviridae	AD, 83	RAS, 83	Epi + sem
GB virus C	Flaviviridae	NAA	No data found	Epi + mol
Hepatitis C virus	Flaviviridae	NAA; AD	No data found	Epi + mol
Zika virus	Flaviviridae	NAA, 188	RCC, 7	Epi + mol + sem
Hepatitis B virus	Hepadnaviridae	NAA; AD	RAS	Epi + mol
Cytomegalovirus	Herpesviridae	NAA	RCC	Epi + mol + sem
Epstein Barr virus	Herpesviridae	NAA	No data found	Epi and semen
Human herpes virus 8	Herpesviridae	NAA	RCC	Epi + mol
Human herpes virus 7	Herpesviridae	NAA	No data found	Unknown
Human herpes virus 6	Herpesviridae	NAA	No data found	Unknown
Human simplex viruses 1 and 2	Herpesviridae	NAA; AD	RCC	Epi + mol + sem
Varicella zoster virus	Herpesviridae	NAA	No data found	Unknown
Mumps virus†	Paramyxoviridae	NAA, 40	RCC, 14	Unknown
Adeno-associated virus	Parvoviridae	NAA	RCC	Unknown
BK virus	Polyomaviridae	NAA	No data found	Unknown
JC virus	Polyomaviridae	NAA	No data found	Unknown
Simian virus 40	Polyomaviridae	NAA	No data found	Unknown
HIV	Retroviridae	NAA; AD	RCC	Epi + mol + sem
Human T-cell lymphoma virus 1†	Retroviridae	No data found	RAS	Epi + mol
Simian foamy virus	Retroviridae	NAA	No data found	Unknown
Chikungunya virus†	Togaviridae	NAA, 30	No data found	Unknown

*Presence of nucleic acid or antigen in semen does not represent the presence of replication-competent or infection-competent virus, which can generally only be demonstrated by isolation and culture of virus. Maximum detection time refers to time from symptom onset (only in viruses that cause acute only, not chronic, infection). A complete table with references is provided in the Technical Appendix). AD, antigen detection; Epi, epidemiologic evidence of sexual transmission; mol, molecular/phylogenetic evidence of sexual transmission; NAA, nucleic acid amplification or detection; RAS, replication in animal system; RCC, replication in cell culture; sem, isolation from semen.  †Data found only in the context of case reports and not case series, case control, or cohort studies.

Given these findings, the following questions need to be addressed: which viruses are shed and remain viable in semen, for how long, and at what concentrations? The answers to these questions have implications for risks for sexual transmission and, therefore, embryonic infection, congenital disease, miscarriage, and effects on epidemiologic and transmission models. The presence of virus in the male reproductive tract may increase the risk for acquisition of sexually transmitted infections and may reduce male fertility through spermatogonial stem cell infection or local inflammation. Infection of spermatozoa could result in transmission of virus-induced mutations to subsequent generations, thereby elevating risks for cancer and other disorders. Indeed, when virus has been detected in human semen, the extent to which virus existence and replication occurs within spermatozoa is unclear (*8*). Not all therapeutics will cross the male reproductive tract–blood barriers, and viruses may persist in semen despite systemic clearance of virus, highlighting the need to consider the male reproductive tract–blood barriers when choosing therapeutic agents in clinical trials. Virus within the male reproductive tract can also be genetically distinct from virus in other compartments, including blood (*9*), which has implications for gene-based vaccines and therapeutics.

The presence of viruses in semen is probably more widespread than currently appreciated, and the absence of virus in genital secretions should not be assumed for traditionally non–sexually transmitted viruses. The investigation of virus detection and persistence in semen across a range of viruses is useful for clinical and public health reasons, in particular for viruses that lead to high mortality or morbidity rates or to epidemics.

Technical Appendix Results of literature search for viruses that are capable of causing viremia and found in human semen.
