# Supplementary material for: The Breadth of Viruses in Human Semen
Source: Emerg Infect Dis. 2017 Nov;23(11):1922–4. doi: 10.3201/eid2311.171049 (PMC5652425; doi:10.3201/eid2311.171049)
Supplement: Technical Appendix — Results of literature search for viruses that are capable of causing viremia and found in human semen. [file 17-1049-Techapp-s1.pdf]

Article DOI: https://doi.org/10.3201/eid2311.171049

# The Breadth of Viruses in Human Semen

## Technical Appendix

**Technical Appendix Table.** Viruses that are capable of causing viremia and found in human semen*

| Virus | Family | Detection in semen, maximum detection time, d | Isolation from semen maximum detection time, d | Evidence for sexual transmission within same cohort |
|---|---|---|---|---|
| Adenoviruses | Adenoviridae | AD (*1*) | RCC | Unknown |
| Transfusion transmitted virus | Anelloviridae | NAA (*2*) | No data found | Unknown |
| Lassa fever virus† | Arenaviridae | NAA, 103 (*3*) | RCC, 20 (*3*) | Unknown |
| Rift Valley fever virus† | Bunyaviridae | NAA, 117 (*4*) | No data found | Unknown |
| Ebola virus | Filoviridae | NAA, 531 (*5*) | RCC, 82 (*6*) | Epi + mol + sem (*7*) |
| Marburg virus† | Filoviridae | AD, 83 (*8*) | RAS, 83 (*8*) | Epi + sem (*9*) |
| GB virus C | Flaviviridae | NAA (*10*) | No data found | Epi + mol (*11*) |
| Hepatitis C virus | Flaviviridae | NAA (*12*); AD (*13*) | No data found | Epi + mol (*14*) |
| Zika virus | Flaviviridae | NAA, 188 (*15*) | RCC, 7 (*16*) | Epi + mol + sem (*17*) |
| Hepatitis B virus | Hepadnaviridae | NAA (*18*); AD (*19*) | RAS (*20*) | Epi + mol (*21*) |
| Cytomegalovirus | Herpesviridae | NAA (*22*) | RCC (*23*) | Epi + mol + sem (*24*) |
| Epstein Barr virus | Herpesviridae | NAA (*22*) | No data found | Epi and semen (*25*) |
| Human herpes virus 8 | Herpesviridae | NAA (*26*) | RCC (*27*) | Epi + mol (*28*) |
| Human herpes virus 7 | Herpesviridae | NAA (*29*) | No data found | Unknown |
| Human herpes virus 6 | Herpesviridae | NAA (*22*) | No data found | Unknown |
| Human simplex viruses 1 and 2 | Herpesviridae | NAA (*22*); AD (*1*) | RCC (*1*) | Epi + mol + sem (*30*) |
| Varicella zoster virus | Herpesviridae | NAA (*22*) | No data found | Unknown |
| Mumps virus† | Paramyxoviridae | NAA, 40 (*31*) | RCC, 14 (*31*) | Unknown |
| Adeno-associated virus | Parvoviridae | NAA (*32*) | RCC (*33*) | Unknown |
| BK virus | Polyomaviridae | NAA (*34*) | No data found | Unknown |
| JC virus | Polyomaviridae | NAA (*34*) | No data found | Unknown |
| Simian virus 40 | Polyomaviridae | NAA (*35*) | No data found | Unknown |
| HIV | Retroviridae | NAA (*36*), AD (*37*) | RCC (*38*) | Epi + mol + sem (*39*) |
| Human T-cell lymphoma virus 1† | Retroviridae | No data found | RAS (*40*) | Epi + mol (*41*) |
| Simian foamy virus | Retroviridae | NAA (*42*) | No data found | Unknown |
| Chikungunya virus† | Togaviridae | NAA, 30 (*43*) | No data found | Unknown |

*Presence of nucleic acid or antigen in semen does not represent the presence of replication-competent or infection-competent virus, which can generally only be demonstration by isolation and culture of virus. Maximum detection time refers to time from symptom onset (only in viruses that cause acute only, not chronic, infection). AD, antigen detection; Epi, Epidemiological evidence of sexual transmission; mol, molecular/phylogenetic evidence of sexual transmission; NAA, nucleic acid amplification or detection; RAS, replication in animal system; RCC, replication in cell culture; sem, isolation from semen. †Data found only in the context of case reports, and not case series, case control, or cohort studies.

## References


1. Kulcsár G, Csata S, Nász I. Investigations into virus carriership in human semen and mouse testicular cells. Acta Microbiol Hung. 1991;38:127–32. PubMed

2. Matsubara H, Michitaka K, Horiike N, Yano M, Akbar SM, Torisu M, et al. Existence of TT virus DNA in extracellular body fluids from normal healthy Japanese subjects. Intervirology. 2000;43:16–9. PubMed

3. Raabe VN, Kann G, Ribner BS, Morales A, Varkey JB, Mehta AK, et al. Favipiravir and ribavirin treatment of epidemiologically linked cases of lassa fever. Clin Infect Dis. 2017;65:1–5.

4. Haneche F, Leparc-Goffart I, Simon F, Hentzien M, Martinez-Pourcher V, Caumes E, et al. Rift Valley fever in kidney transplant recipient returning from Mali with viral RNA detected in semen up to four months from symptom onset, France, autumn 2015. Euro Surveill. 2016;21:30222–4. PubMed

5. Diallo B, Sissoko D, Loman NJ, Bah HA, Bah H, Worrell MC, et al. Resurgence of Ebola virus disease in guinea linked to a survivor with virus persistence in seminal fluid for more than 500 days. Clin Infect Dis. 2016;63:1353–6. PubMed

6. Rodriguez LL, De Roo A, Guimard Y, Trappier SG, Sanchez A, Bressler D, Et al. Persistence and genetic stability of Ebola virus during the outbreak in Kikwit, Democratic Republic of the Congo, 1995. J Infect Dis. 1999 Feb;179 Suppl 1(S1):S170–6.

7. Mate SE, Kugelman JR, Nyenswah TG, Ladner JT, Wiley MR, Cordier-Lassalle T, et al. Molecular evidence of sexual transmission of Ebola virus. N Engl J Med. 2015;373:2448–54. PubMed

8. Siegert R, Shu HL, Slenczka W. Nachweis des “Marburg-Virus” beim Patienten [in German]. Dtsch Med Wochenschr. 1968;93:616–9. PubMed

9. Martini GA, Schmidt HA. Spermatogenic transmission of the “Marburg virus”. (Causes of “Marburg simian disease”) [in German]. Klin Wochenschr. 1968;46:398–400. PubMed

10. Semprini AE, Persico T, Thiers V, Oneta M, Tuveri R, Serafini P, et al. Absence of hepatitis C virus and detection of hepatitis G virus/GB virus C RNA sequences in the semen of infected men. J Infect Dis. 1998;177:848–54. PubMed

11. Sarrazin C, Roth WK, Zeuzem S. Heterosexual transmission of GB virus-C/hepatitis G virus infection. Eur J Gastroenterol Hepatol. 1997;9:1117–20. PubMed

12. Leruez-Ville M, Kunstmann J-M, De Almeida M, Rouzioux C, Chaix M-L. Detection of hepatitis C virus in the semen of infected men. Lancet. 2000;356:42–3. PubMed

13. Kotwal GJ, Rustgi VK, Baroudy BM. Detection of hepatitis C virus-specific antigens in semen from non-A, non-B hepatitis patients. Dig Dis Sci. 1992;37:641–4. PubMed

14. Terrault NA, Dodge JL, Murphy EL, Tavis JE, Kiss A, Levin TR, et al. Sexual transmission of hepatitis C virus among monogamous heterosexual couples: the HCV partners study. Hepatology. 2013;57:881–9. PubMed

15. Nicastri E, Castilletti C, Liuzzi G, Iannetta M, Capobianchi MR, Ippolito G. Persistent detection of Zika virus RNA in semen for six months after symptom onset in a traveller returning from Haiti to Italy, February 2016. Euro Surveill. 2016;21:30314–4. PubMed

16. Jang H-C, Park WB, Kim UJ, Chun JY, Choi S-J, Choe PG, et al. First imported case of Zika virus infection into Korea. J Korean Med Sci. 2016;31:1173–7. PubMed

17. D'Ortenzio E, Matheron S, Yazdanpanah Y, de Lamballerie X, Hubert B, Piorkowski G, et al. Evidence of sexual transmission of Zika virus. N Engl J Med. 2016;374:2195–8. PubMed

18. Fei QJ, Yang XD, Ni WH, Pan CS, Huang XF. Can hepatitis B virus DNA in semen be predicted by serum levels of hepatitis B virus DNA, HBeAg, And HBsAg In chronically infected men from infertile couples? Andrology. 2015;3:506–11.

19. Mansour W, Lemoine M, Neri Pinto F, Llabador de Royer MA, Le Gal F, Yazbeck C, et al. Markers of hepatitis delta virus infection can be detected in follicular fluid and semen. J Clin Virol. 2014;61:279–81. PubMed

20. Scott RM, Snitbhan R, Bancroft WH, Alter HJ, Tingpalapong M. Experimental transmission of hepatitis B virus by semen and saliva. J Infect Dis. 1980;142:67–71. PubMed

21. Huo TI, Wu JC, Huang YH, Yang UC, Sheen IJ, Chang FY, et al. Evidence of transmission of hepatitis B virus to spouses from sequence analysis of the viral genome. J Gastroenterol Hepatol. 1998;13:1138–42. PubMed

22. Neofytou E, Sourvinos G, Asmarianaki M, Spandidos DA, Makrigiannakis A. Prevalence of human herpes virus types 1-7 in the semen of men attending an infertility clinic and correlation with semen parameters. Fertil Steril. 2009;91:2487–94. PubMed

23. Bresson JL, Clavequin MC, Mazeron MC, Mengelle C, Scieux C, Segondy M, et al.; Fédération Française des CECOS. Risk of cytomegalovirus transmission by cryopreserved semen: a study of 635 semen samples from 231 donors. Hum Reprod. 2003;18:1881–6. PubMed

24. Handsfield HH, Chandler SH, Caine VA, Meyers JD, Corey L, Medeiros E, et al. Cytomegalovirus infection in sex partners: evidence for sexual transmission. J Infect Dis. 1985;151:344–8. PubMed

25. Thomas R, Macsween KF, McAulay K, Clutterbuck D, Anderson R, Reid S, et al. Evidence of shared Epstein-Barr viral isolates between sexual partners, and low level EBV in genital secretions. J Med Virol. 2006;78:1204–9. PubMed

26. Viviano E, Vitale F, Ajello F, Perna AM, Villafrate MR, Bonura F, et al. Human herpesvirus type 8 DNA sequences in biological samples of HIV-positive and negative individuals in Sicily. AIDS. 1997;11:607–12. PubMed

27. Bagasra O, Patel D, Bobroski L, Abbasi JA, Bagasra AU, Baidouri H, et al. Localization of human herpesvirus type 8 in human sperms by in situ PCR. J Mol Histol. 2005;36:401–12. PubMed

28. Kouri V, Marini A, Doroudi R, Nambiar S, Rodriguez ME, Capo V, et al. Molecular epidemiology of Kaposi's sarcoma herpesvirus (KSHV) in Cuban and German patients with Kaposi's sarcoma (KS) and asymptomatic sexual contacts. Virology. 2005;337:297–303. PubMed

29. Michou V, Liarmakopoulou S, Thomas D, Tsimaratou K, Makarounis K, Constantoulakis P, et al. Herpes virus infected spermatozoa following density gradient centrifugation for IVF purposes. Andrologia. 2012;44:174–80. PubMed

30. Moore DE, Ashley RL, Zarutskie PW, Coombs RW, Soules MR, Corey L. Transmission of genital herpes by donor insemination. JAMA. 1989;261:3441–3. PubMed

31. Jalal H, Bahadur G, Knowles W, Jin L, Brink N. Mumps epididymo-orchitis with prolonged detection of virus in semen and the development of anti-sperm antibodies. J Med Virol. 2004;73:147–50. PubMed

32. Erles K, Rohde V, Thaele M, Roth S, Edler L, Schlehofer JR. DNA of adeno-associated virus (AAV) in testicular tissue and in abnormal semen samples. Hum Reprod. 2001;16:2333–7. PubMed

33. Schlehofer JR. Adeno-vssociated Virus (AAV) in semen and testis: a role in infertility? An overview. Andrologia. 2004;14:317–9.

34. Comar M, Zanotta N, Croci E, Murru I, Marci R, Pancaldi C, et al. Association between the JC polyomavirus infection and male infertility. PLoS One. 2012;7:e42880. PubMed

35. Martini F, Iaccheri L, Lazzarin L, Carinci P, Corallini A, Gerosa M, et al. SV40 early region and large T antigen in human brain tumors, peripheral blood cells, and sperm fluids from healthy individuals. Cancer Res. 1996;56:4820–5. PubMed

36. Mermin JH, Holodniy M, Katzenstein DA, Merigan TC. Detection of human immunodeficiency virus DNA and RNA in semen by the polymerase chain reaction. J Infect Dis. 1991;164:769–72. PubMed

37. Homsy J, Thomson-Honnebier GA, Cheng-Mayer C, Levy JA. Detection of human immunodeficiency virus (HIV) in serum and body fluids by sequential competition ELISA. J Virol Methods. 1988;19:43–56. PubMed

38. Van Voorhis BJ, Martinez A, Mayer K, Anderson DJ. Detection of human immunodeficiency virus type 1 in semen from seropositive men using culture and polymerase chain reaction deoxyribonucleic acid amplification techniques. Fertil Steril. 1991;55:588–94. PubMed

39. Butler DM, Delport W, Kosakovsky Pond SL, Lakdawala MK, Cheng PM, Little SJ, et al. The origins of sexually transmitted HIV among men who have sex with men. Sci Transl Med. 2010 Feb 10;2(18):18re1–18re1.

40. Iwahara Y, Takehara N, Kataoka R, Sawada T, Ohtsuki Y, Nakachi H, et al. Transmission of HTLV-I to rabbits via semen and breast milk from seropositive healthy persons. Int J Cancer. 1990;45:980–3. PubMed

41. Iga M, Okayama A, Stuver S, Matsuoka M, Mueller N, Aoki M, et al. Genetic evidence of transmission of human T cell lymphotropic virus type 1 between spouses. J Infect Dis. 2002;185:691–5. PubMed

42. Boneva RS, Switzer WM, Spira TJ, Bhullar VB, Shanmugam V, Cong ME, et al. Clinical and virological characterization of persistent human infection with simian foamy viruses. AIDS Res Hum Retroviruses. 2007;23:1330–7. PubMed

43. Bandeira AC, Campos GS, Rocha VFD, Souza BS, Soares MBP, Oliveira AA, et al. Prolonged shedding of Chikungunya virus in semen and urine: A new perspective for diagnosis and implications for transmission. IDCases. 2016;6:100–3. PubMed
